# Hyperhidrosis Prevalence and Demographical Characteristics in Dermatology Outpatients in Shanghai and Vancouver

**DOI:** 10.1371/journal.pone.0153719

**Published:** 2016-04-22

**Authors:** Yudan Liu, Rayeheh Bahar, Sunil Kalia, Rachel Yuanshen Huang, Arlie Phillips, Mingwan Su, Sen Yang, Xuejun Zhang, Pingyu Zhou, Youwen Zhou

**Affiliations:** 1 Shanghai Skin Disease Hospital, Clinical School of Anhui Medical University, Shanghai, China; 2 Department of Dermatology and Skin Science, University of British Columbia, Vancouver, BC, Canada; 3 Molecular Medicine Lab and Chieng Genomics Center, Vancouver Coastal Health Research Institute, Vancouver, Canada; 4 Department of Dermatology, First Affiliated Hospital, Anhui Medical University, Hefei, Anhui, China; 5 Sexually Transmitted Disease Institute, Shanghai Skin Disease Hospital, Shanghai, China; The University of Hong Kong, HONG KONG

## Abstract

**Background:**

There is a wide variation in the reported prevalence of primary hyperhidrosis in the literature. Further, it is unknown if primary hyperhidrosis is a lifelong condition, or if demographical factors influence hyperhidrosis prevalence.

**Objectives:**

This study aims to examine the prevalence of hyperhidrosis in multiple ethnic groups from two ethnically diverse cities and to determine if the prevalence of primary hyperhidrosis changes according to age, gender, ethnicity, body mass index, and geographical locations.

**Methods:**

In total, 1010 consecutive subjects attending dermatology outpatient clinics in Shanghai Skin Disease Hospital and 1018 subjects in Skin Care Center of Vancouver General Hospital were invited to fill out a questionnaire on their presenting concerns, demographical information, and sweating symptoms. The subjects were then classified to have primary hyperhidrosis using the criteria of International Hyperhidrosis Society, late-onset hyperhidrosis, or no-hyperhidrosis. The prevalence of primary HH and late-onset HH was calculated for the entire study population and in subgroups stratified according to age of examination, sex, ethnicity, presenting diagnosis, body mass index, and specific study cities. Multivariate logistic regression analyses were performed to assess the impact of these factors on HH prevalence.

**Results:**

The prevalence of primary hyperhidrosis is very similar in Shanghai and in Vancouver, at 14.5% and 12.3% respectively. In addition, 4.0% of subjects in Shanghai and 4.4% subjects in Vancouver suffer from late-onset HH. Primary HH has highest prevalence in those younger than 30 years of age, decreasing dramatically in later years. Caucasian subjects are at least 2.5 times more likely to develop axillary hyperhidrosis compared to Chinese subjects. Obesity does not have much influence on primary HH presentation, although it does increase significantly the development of late-onset HH. Finally, there is no major difference of hyperhidrosis between Chinese subjects in Shanghai and Vancouver.

**Limitations:**

The data were gathered according to patients’ self-reports only and the sample size was relatively small in some groups after stratification for gender, ethnicity and age.

**Conclusion:**

Prevalence of primary HH and late-onset HH is similar in dermatology outpatients independent of geographical locations. However, certain specific HH subtypes can show great variations according to ethnicity, age, body mass index and sex.

## Introduction

Hyperhidrosis (HH) is a condition characterized by excessive sweating without the usual triggers such as mental, physiological or thermal stimuli [1]. There are two broad categories of hyperhidrosis, secondary and primary. Secondary hyperhidrosis occur with an underlying medical condition, such as chronic infections and other catabolic conditions [2]. In contrast, primary hyperhidrosis (primary HH) occurs in a symmetrical fashion with an age of onset less than 30 years and without underlying medical conditions. While the significant negative impact of primary HH is well known, especially in developed countries [3], there have been large variations in the prevalence of primary HH in various reports, ranging from 2.8% in the United States [4], to 16.3% in Germany [5] and 12.8% in Japan [6], the reasons for the discrepancies are not known. However, the demographical and geographical aspects of various studies differ from one study to the next. Further, the criteria used for the diagnosis and classification of HH also varied from study to study. Finally, the methodology used for assessing the prevalence of primary HH also differed. In order to examine the prevalence of primary HH in different geographical regions, and assess the impact of demographical parameters on HH and various subtypes, we conducted a two- center questionnaire-based study in dermatology outpatients in Vancouver, Canada and Shanghai, China using the same questionnaire by assessing one thousand consecutive subjects in each center. The results showed that despite the differences present in the ethnical composition, average age, specific reasons for presenting to the dermatology clinics, and differing levels of hyperhidrosis awareness that exist between the two study centers, the prevalence of primary HH is similar between these two centers. Multivariate analyses showed that the presentation and prevalence of primary HH and subtypes do vary with ethnicity, age, sex, and body mass index.

## Methods

### Overall Study Design

This is a two-center study using the same methodology and questionnaire, using the criteria of International Hyperhidrosis Society for the definition of primary HH [7]. The Vancouver center is located in the outpatient clinic at the Department of Dermatology and Skin Science of University of British Columbia, a tertiary referral center for patients with medical, surgical and cosmetic dermatology concerns. The Shanghai center is located in the outpatient clinic of Shanghai Skin Disease Hospital, a primary care medical dermatology and venereology center in Shanghai. This study was approved by the Clinical Ethics Board of University of British Columbia. Written informed consents were obtained from all participants.

### Study Populations

The study took place from June 2014 to March 2015. In total 1038 subjects in Vancouver and 1010 in Shanghai were recruited. 98% the subjects in Vancouver and 100% subjects in Shanghai completed the questionnaires. In Vancouver, 95% filled the questionnaire in English, with 5% remaining subjects filling out the Chinese version of the same questionnaire. In Shanghai, all subjects completed the questionnaire in Chinese.

### The hyperhidrosis questionnaire and data collection

The questionnaire contained the following components: ethnicity, age, sex, height, body weight, reasons for presenting for dermatological assessment, symptoms related to day-time sweating, night time sweating, negative impact of sweating, anxiety and depression as recalled within the last two weeks prior to the presentation. The severity was based on frequency of unprovoked sweating: on almost every day (score 3), more than half of the days (score 2), less than half of the days (score 1), or not at all (score 0). Finally, the relative severity of sweating affecting various body sites was also collected according to the following scale: no sweating (score 0), mild sweating (damp, no visible drops, score 1); moderate sweating (visible sweat drops but not dripping sweats, score 2), or severe (dripping sweating, score 3). The assignments of HH subtypes are according to the following criteria: primary HH refers to symmetrical sweating with the age of onset less than 30 years in the absence of mental, physiological or thermal triggers [7]. Since most subjects report sweating affecting multiple body sites, assignment of anatomic subtypes of primary HH is according to the body sites most severely affected. For patients who suffer from HH with no obvious reasons but have age of onset greater than 30 years, a definition of late-onset hyperhidrosis (LO-HH) is used. The “prevalence” calculations had excluded patients who presented for hyperhidrosis.

To ensure that the questionnaires were filled out properly, every questionnaire returned was checked by a study assistant and any unclear answer or incompletely filled out item was checked and verified with the patient during the same visit. The classifications of presenting diagnoses are the same in both centers for the most common twelve categories. The remaining cases are grouped into “miscellaneous” category. The diagnoses were assigned by the dermatologists assessing the subjects in the clinics.

### Ethnicities

For evaluation of the effect of ethnicity on the HH prevalence, we divided the patients into four main ethnic groups: Caucasians (Cau), Chinese (Chi), South-Southeast Asians (SSEA), and others.

### Statistical Analysis

The data analysis was performed with Microsoft Excel, SPSS 22 and R Software. The point prevalence of HH was measured for the whole subject population excluding those who presented because of hyperhidrosis to remove referral bias. Chi-square and logistic regression models were used and P-value <0.05 was considered as significant.

## Results

### General Demographic Information

Due to the differences of the referral patterns, there are significant differences between the two study centers in ethnicity, age, gender, body mass index, and primary dermatological diagnoses (**Table 1**). The Shanghai cohort consists of Chinese only and has higher female ratio (62.5%), a younger presenting age (34.5 years) and lower body mass index (BMI = 21.7) compared with the Vancouver cohort (Caucasians, Chinese and South Southeast Asians; 55.7% female, 45.1 years of average age, and an average BMI of 23.9, p values are significant statistically for all parameters). The presenting diagnoses are also very different, with the top five diagnoses for Vancouver being benign tumors (22.3%), eczematous conditions (16.5%), dysplastic/in situ or invasive malignancies (10.2%), hyperhidrosis (8%) and pigmentations disorders (7.8%); whereas the most common diagnoses in Shanghai center being eczematous disorders (32.4%), skin infections (23%), acne (19.5%), hair disorders (11%), and psoriasis (7.4%).

**Table 1:** Demographics and Presenting Diagnoses in Outpatient Dermatology Clinics.

Study Center		Vancouver		Shanghai		*P*- value
		Number	%	Number	%	
**Subjects (Total)**		1018	100	1010	100	
**Gender**	** **					
** **	**Male**	451	44.3	379	37.5	0.0018
	**Female**	567	55.7	631	62.5	
**Ethnicity**	** **					
	**Caucasians**	359	35.3	0	0	1.80E-192
** **	**Chinese**	488	47.9	1010	100	
** **	**SSEA**	107	10.5	0	0	
** **	**Others**	64	6.3	0	0	
**Age (yr)**	** **	45.1	-	34.5	-	2.10E-43
**BMI**	** **	23.9	-	21.7	-	4.80E-27
**Presenting diagnosis**						
** **	**Acne**	46	4.5	197	19.5	6.50E-26
** **	**ECZ**	168	16.5	327	32.4	5.00E-17
** **	**Hair Ds**	36	3.5	11	11	2.40E-04
** **	**HH**	81	8	0	0	2.50E-20
** **	**INF**	42	4.1	232	23	1.10E-36
** **	**MISC**	117	11.5	140	13.9	0.109
** **	**PIG-MISC**	79	7.8	5	0.5	1.30E-16
** **	**PIG-VIT**	36	3.5	6	0.6	3.00E-06
** **	**PSO**	34	3.3	75	7.4	4.00E-05
** **	**ROS**	25	2.5	2	0.2	9.00E-06
** **	**Scars**	23	2.3	1	0.1	7.00E-06
** **	**Tumor-BN**	227	22.3	13	1.3	4.00E-51
** **	**Tumor-D/IS/M**	104	10.2	1	0.1	2.20E-25

Abbreviations: SSEA: South and Southeast Asians; BMI: Bodymass index; ECZ: eczematous conditions; HH: hyperhidrosis; INF: infections; MISC: miscellaneous; PIG-MISC: miscellaneous pigmentation disorders (excluding vitiligo); PIG-VIT: pigmentation disorder-vitiligo; PSO: psoriasis; ROS: Rosacea, Tumor-BN: benign tumors; Tumor-DISM: dysplastic, in situ, or invasive malignancies.

### Awareness and prevalence of hyperhidrosis in Shanghai and Vancouver dermatology outpatient clinics

In Vancouver study center, 8% of the subjects presented with hyperhidrosis as the primary concern, as compared with no such patients in the Shanghai center (**Table 1**, p = 2.5E-20), indicating there is a low level of awareness of HH in Shanghai. However, the prevalence of total hyperhidrosis (primary HH and late-onset HH) is similar between Vancouver and Shanghai cohorts (16.7% and 18.4%, respectively, p >0.05, **Table 2**). Similarly, there was no difference between these two centers in late-onset HH (4.0% vs 4.4% respectively, p >0.05), primary HH (14.5% vs 12.3% respectively, p>0.05, **Table 2**). The average HH severity score and negative impact scores are also similar between Shanghai and Vancouver centers (**S1 Fig**), suggesting that the low awareness of HH in Shanghai cannot be explained by factors such as prevalence, severity or degree of negative impact on daily activities.

**Table 2:** Prevalence of Hyperhidrosis in Various Ethnic Groups of Outpatient Dermatology Patients in Vancouver and Shanghai.

Groups	HH-Total (%)	Primary HH				LO-HH(%)
		All Pri HH (%)	AH (%)	PPH (%)	GFH (%)	
**Shanghai**						
**Chinese**	18.4	14.5	2.5	5.8	6.1 ^*a^	4.0
**Vancouver**						
**Total**	16.7	12.3	4.4	5.7	2.2	4.4
** Chinese**	15.4	11.9	3.1^*b^	6.6	2.3^*a^	3.5^*c^
**Caucasian**	18.1	11.7	5.8^*b^	4.2	1.7	6.4^*c^
**SSEA**	15.0	13.1	4.7	7.5	0.9	1.9
**Others**	21.9	17.2	6.3	4.7	6.3	4.7

Abbreviations: SSEA: South and Southeast Asians; AH: axillary hyperhidrosis, PPH: palmoplantar hyperhidrosis; GFH: generalized and facial hyperhidrosis; LO-HH: late-onset hyperhidrosis (>30 years of age).

*a: p<0.05 between these two groups.

*b: p<0.05 between these two groups.

*c: p <0.05 between these two groups.

### Ethnicity and prevalence of hyperhidrosis

Since the Vancouver cohort contained subjects from multiple ethnic origins, the prevalence and anatomic patterns of hyperhidrosis can be compared among various ethnic groups. As shown in **Table 2,** the prevalence of total HH and primary HH did not differ significantly among the three main ethnic groups. However, the prevalence of anatomical subtypes of HH does differ significantly among these groups. Chinese subjects are 2.5 to 5 times less likely to suffer from axillary HH compared with Caucasians (p <0.05, **Tables 2–5**). There is a trend of less hyperhidrosis of PPH and GFH subtypes in Chinese population, although individually these differences did not reach the level of significance.

**Table 3:** Prevalence of HH and subtypes in male and female patients in dermatology outpatients in Vancouver and Shanghai.

		HH-Total (%)	Primary HH				LO-HH (%)
			All Pri HH (%)	AH (%)	PPH (%)	GFH (%)	
**Vancouver**							
	**Male**	14.4	11.5	2.9	6.7	2.0	2.9
	**Female**	18.5	12.9	5.6	4.9	2.3	5.6
	***p***	*0*.*081*	*0*.*517*	***0*.*033***	*0*.*242*	*0*.*746*	***0*.*033***
**V-Cau**							
	**Male**	16.4	12.6	6.3	5.0	1.3	3.8
	**Female**	19.6	11.1	5.5	3.5	2.0	8.5
	***p***	*0*.*430*	*0*.*657*	*0*.*761*	*0*.*479*	*0*.*583*	*0*.*068*
**V-SSEA**							
	**Male**	15.4	11.5	1.9	9.6	0.0	3.8
	**Female**	14.5	14.5	7.3	5.5	1.8	0.0
	***p***	*0*.*874*	*0*.*675*	*0*.*201*	*0*.*403*	*0*.*338*	*0*.*141*
**V-Chi**							
	**Male**	12.3	10.0	0.9	6.8	2.3	2.3
	**Female**	17.8	13.4	4.8	6.3	2.2	4.5
	***p***	*0*.*093*	*0*.*258*	***0*.*013***	*0*.*815*	*0*.*969*	*0*.*193*
**SH-Chi**							
	**Male**	23.0	18.5	3.2	7.1	8.2	4.5
	**Female**	15.7	12.0	2.1	5.1	4.9	3.6
	***p***	***0*.*004***	***0*.*005***	*0*.*274*	*0*.*178*	***0*.*036***	*0*.*508*

Abbreviations: V-Cau: Caucasians in Vancouver; V-SSEA: South and Southeast Asians in Vancouver; V-Chi: Chinese in Vancouver; SH-Chi: Chinese in Shanghai; AH: axillary hyperhidrosis, PPH: palmoplantar hyperhidrosis; GFH: generalized and facial hyperhidrosis; LO-HH: late-onset hyperhidrosis (>30 years of age).

**Table 4:** Influence of body mass index on the prevalence of hyperhidrosis in dermatology outpatient clinics in Shanghai and Vancouver.

		HH-Total	Pri HH	AH	PPH	GFH	LO-HH
**Vancouver (All subjects)**							
	**BMI >24.9**	20.50%	12.80%	5.10%	4.80%	2.80%	7.70%
	**BMI <24.9**	14.80%	12.00%	4.10%	6.10%	1.80%	2.70%
	***p***	***0*.*011***	*0*.*363*	*0*.*231*	*0*.*205*	*0*.*147*	***0*.*000***
**V-Cau**							
	**BMI >24.9**	20.90%	12.10%	5.50%	4.90%	1.60%	8.80%
	**BMI <24.9**	15.60%	11.60%	6.40%	3.50%	1.70%	4.00%
	***p***	*0*.*100*	*0*.*439*	*0*.*366*	*0*.*245*	*0*.*475*	***0*.*035***
**V-SSEA**							
	**BMI >24.9**	18.60%	14.00%	4.70%	9.30%	0.00%	4.70%
	**BMI <24.9**	11.10%	11.10%	4.80%	4.80%	1.60%	0.00%
	***p***	*0*.*141*	*0*.*332*	*0*.*490*	*0*.*180*	*0*.*206*	***0*.*043***
**V-Chi**							
	**BMI >24.9**	19.30%	12.80%	4.60%	3.70%	4.60%	6.40%
	**BMI <24.9**	14.40%	11.70%	2.70%	7.50%	1.60%	2.70%
	***p***	*0*.*109*	*0*.*377*	*0*.*155*	*0*.*080*	***0*.*033***	***0*.*031***
**SH-Chi**							
	**BMI >24.9**	22.00%	13.60%	2.30%	3.80%	7.60%	8.30%
	**BMI <24.9**	17.90%	14.60%	2.50%	6.20%	5.90%	3.30%
	***p***	*0*.*130*	*0*.*387*	*0*.*436*	*0*.*141*	*0*.*231*	***0*.*003***

Abbreviations: V-Cau: Caucasians in Vancouver; V-SSEA: South and Southeast Asians in Vancouver; V-Chi: Chinese in Vancouver; SH-Chi: Chinese in Shanghai; Pri-HH: primary hyperhidrosis, AH: axillary hyperhidrosis, PPH: palmoplantar hyperhidrosis; GFH: generalized and facial hyperhidrosis; LO-HH: late-onset hyperhidrosis (>30 years of age).

**Table 5:** Multivariate Regression Analysis on Various Parameters Associated with Hyperhidrosis.

	Primary Hyperhidrosis																Late-Onset Hyperhidrosis			
	Total				AH				PPH				GFH				LO-HH			
	OR	95%CI (L)	95% CI (U)	Sig.	OR	95% CI (L)	95% CI (U)	Sig.	OR	95% CI (L)	95% CI (U)	Sig.	OR	95% CI (L)	95% CI (U)	Sig.	OR	95% CI (L)	95%	Sig.
																			CI(U)	
**Diagnosis**																				
**MISC**	1.0	1.0	1.0	1.000	1.0	1.0	1.0	1.000	1.0	1.0	1.0	1.000	1.0	1.0	1.0	1.000	1.0	1.0	1.0	1.000
**Acne**	1.2	0.7	2.0	0.518	2.1	0.7	6.5	0.198	0.6	0.3	1.5	0.293	1.4	0.6	2.9	0.404	0.5	0.1	1.9	0.343
**ECZ**	1.1	0.7	1.8	0.571	2.0	0.7	5.7	0.172	1.3	0.7	2.6	0.437	0.6	0.3	1.3	0.214	0.5	0.2	1.0	0.057
**Hair DS**	0.6	0.2	1.8	0.357	2.2	0.5	10.0	0.304	0.3	0.0	2.7	0.309	0.0	0.0	.^b^	0.998	1.6	0.5	5.5	0.467
**HH**	.^a^	.^a^	.^a^	.^a^	.^a^	.^a^	.^a^	.^a^	.^a^	.^a^	.^a^	.^a^	.^a^	.^a^	.^a^	.^a^	.^a^	.^a^	.^a^	.^a^
**INF**	1.2	0.7	2.1	0.444	2.0	0.6	6.3	0.258	1.3	0.6	2.7	0.537	0.8	0.4	1.8	0.635	0.9	0.4	2.0	0.863
**PIG-MISC**	1.0	0.4	2.5	0.921	1.0	0.2	5.7	0.958	2.0	0.7	5.8	0.199	0.0	0.0	.^b^	0.998	0.4	0.1	1.7	0.202
**PIG-VIT**	*3*.*2*	*1*.*4*	*7*.*5*	*0*.*007*	2.7	0.6	12.4	0.197	*5*.*1*	*1*.*8*	*14*.*7*	*0*.*003*	0.7	0.1	6.1	0.760	0.6	0.1	2.6	0.454
**PSO**	1.3	0.6	2.6	0.476	*3*.*5*	*1*.*0*	*12*.*1*	*0*.*049*	0.7	0.2	2.4	0.527	1.0	0.3	2.9	0.931	0.9	0.3	2.1	0.730
**ROS**	1.8	0.6	5.9	0.311	2.9	0.5	16.7	0.228	.^a^	.^a^	.^a^	.^a^	3.3	0.6	17.3	0.156	1.8	0.5	6.3	0.340
**Scar**	1.2	0.4	3.8	0.804	1.4	0.2	13.3	0.750	2.0	0.5	8.2	0.326	.^a^	.^a^	.^a^	.^a^	0.9	0.1	7.9	0.918
**Tumor-BN**	1.3	0.7	2.3	0.405	1.6	0.5	4.8	0.428	1.3	0.6	3.1	0.503	1.2	0.5	3.3	0.694	0.6	0.3	1.3	0.205
**Tumor-DISN**	0.7	0.3	1.7	0.430	0.9	0.2	4.3	0.933	1.2	0.3	4.2	0.774	.^a^	.^a^	.^a^	.^a^	0.3	0.1	0.9	0.033
**Gender**																				
**M**	1.0	1.0	1.0	1.000	1.0	1.0	1.0	1.000	1.0	1.0	1.0	1.000	1.0	1.0	1.0	1.000	1.0	1.0	1.0	1.000
**F**	*0*.*8*	*0*.*6*	*1*.*0*	*0*.*049*	1.3	0.8	2.2	0.292	*0*.*7*	*0*.*4*	*1*.*0*	*0*.*036*	0.7	0.4	1.0	0.077	*1*.*8*	*1*.*1*	*2*.*9*	*0*.*020*
**BMI**																				
**< = 24.9**	1.0	1.0	1.0	1.000	1.0	1.0	1.0	1.000	1.0	1.0	1.0	1.000	1.0	1.0	1.0	1.000	1.0	1.0	1.0	1.000
**>24.9**	1.2	0.9	1.8	0.220	1.2	0.7	2.2	0.506	0.9	0.5	1.6	0.800	1.7	1.0	3.1	0.069	*2*.*2*	*1*.*3*	*3*.*6*	*0*.*003*
**Ethnicity**																				
**Cau**	1.0	1.0	1.0	1.000	1.0	1.0	1.0	1.000	1.0	1.0	1.0	1.000	1.0	1.0	1.0	1.000	1.0	1.0	1.0	1.000
**CHI-V**	0.7	0.4	1.2	0.172	*0*.*4*	*0*.*2*	*0*.*8*	*0*.*012*	1.1	0.5	2.2	0.802	1.1	0.4	3.1	0.846	0.5	0.3	1.1	0.073
**CHI-SH**	0.6	0.4	1.0	0.055	*0*.*2*	*0*.*1*	*0*.*4*	*0*.*000*	0.8	0.4	1.7	0.572	2.2	0.8	5.9	0.116	1.0	0.5	2.1	0.946
**Others**	1.0	0.4	2.2	0.972	0.7	0.2	2.2	0.515	0.7	0.2	2.8	0.639	2.9	0.7	11.6	0.124	0.8	0.2	3.1	0.788
**SSEA**	0.7	0.4	1.5	0.387	0.5	0.2	1.6	0.263	1.1	0.4	3.2	0.792	0.5	0.1	4.2	0.512	0.3	0.1	1.3	0.107
**Age Group**																				
**> 60 yrs**	1.0	1.0	1.0	1.000	1.0	1.0	1.0	1.000	1.0	1.0	1.0	1.000	1.0	1.0	1.0	1.000	1.0	1.0	1.0	1.000
**<30 yrs**	*6*.*4*	*4*.*1*	*10*.*1*	*0*.*000*	*3*.*9*	*1*.*8*	*8*.*7*	*0*.*001*	*5*.*9*	*3*.*2*	*10*.*9*	*0*.*000*	*7*.*2*	*2*.*7*	*19*.*5*	*0*.*000*	.^a^	.^a^	.^a^	.^a^
**30–60 yrs**	*2*.*5*	*1*.*6*	*3*.*9*	*0*.*000*	*2*.*9*	*1*.*3*	*6*.*1*	*0*.*006*	1.5	0.8	2.9	0.251	*4*.*0*	*1*.*5*	*10*.*8*	*0*.*005*	*0*.*5*	*0*.*3*	*0*.*8*	*0*.*004*

Abbreviations: Cau: Caucasians; CHI-V: Chinese in Vancouver; CHI-SH: Chinese in Shanghai; SSEA: South and Southeast Asians; ECZ: eczematous conditions; HH: hyperhidrosis; INF: infections; MISC: miscellaneous; PIG-MISC: miscellaneous pigmentation disorders (excluding vitiligo); PIG-VIT: pigmentation disorder-vitiligo; PSO: psoriasis; ROS: Rosacea, Tumor-BN: benign tumors; Tumor-DISM: dysplastic, in situ, or invasive malignancies. AH: axillary hyperhidrosis, PPH: palmoplantar hyperhidrosis; GFH: generalized and facial hyperhidrosis; M: Male; F: Females; BMI: Body mass index; OR: odds ratio; yrs: years old; CI (L) and CI (U): lower and upper confidence intervals; Sig: p value with logistic regression analysis.

a: 0, thus no interval or p can be calculated.

b: no upper limit of the interval is calculated statistically due to the since the OR is “0”.

### Geographical influence on HH prevalence

Since the ethnic compositions in Vancouver and Shanghai are quite different, analysis focusing on the Chinese subjects in Vancouver and in Shanghai may be more relevant for assessing potential influence of geographical locations on prevalence of hyperhidrosis. As seen in **Table 2**, there is no significant difference between Chinese in Vancouver and Chinese in Shanghai in the prevalence of total HH, primary HH, axillary HH, palmoplantar HH, and late-onset HH. In single variate analysis, the Chinese in Shanghai has higher generalized and facial hyperhidrosis compared with the Chinese in Vancouver (**Table 2**). However, when taking into account the age and gender differences between these centers, this prevalence difference is no longer apparent based on multivariate analysis (**Table 5**).

### Gender and prevalence of hyperhidrosis

In general, based on single variate analysis, there is a strong trend for females to have higher prevalence of axillary HH and late-onset HH compared with males (**Table 3**), although there are some variations among different ethnic groups. When taking into consideration of potential co-variables in multivariate analysis (**Table 5**), gender has a mild effect on total hyperhidrosis prevalence (OR 0.8), although females having much higher prevalence of late-onset HH (OR = 1.8, p = 0.02), and lower prevalence of palmoplantar HH (OR = 0.7, p = 0.036), and total HH (OR = 0.8, p = 0.049) compared with males.

### Obesity and hyperhidrosis prevalence

Since obesity can be associated with higher physiological stress on the body, and can be associated with increased sweating [8], it could be speculated that increased body mass index (>24.9 classified as obese) may be associated with higher prevalence of primary HH as well. As shown in **Tables 4** and **5**, high BMI as expected increased significantly the prevalence of late-onset HH (OR = 2.2, p = 0.003). However, it does not have any measurable impact on the prevalence of primary HH and its subtypes, suggesting late-onset HH has a different etiology compared with primary HH.

### Hyperhidrosis prevalence varies with age

**Fig 1:**
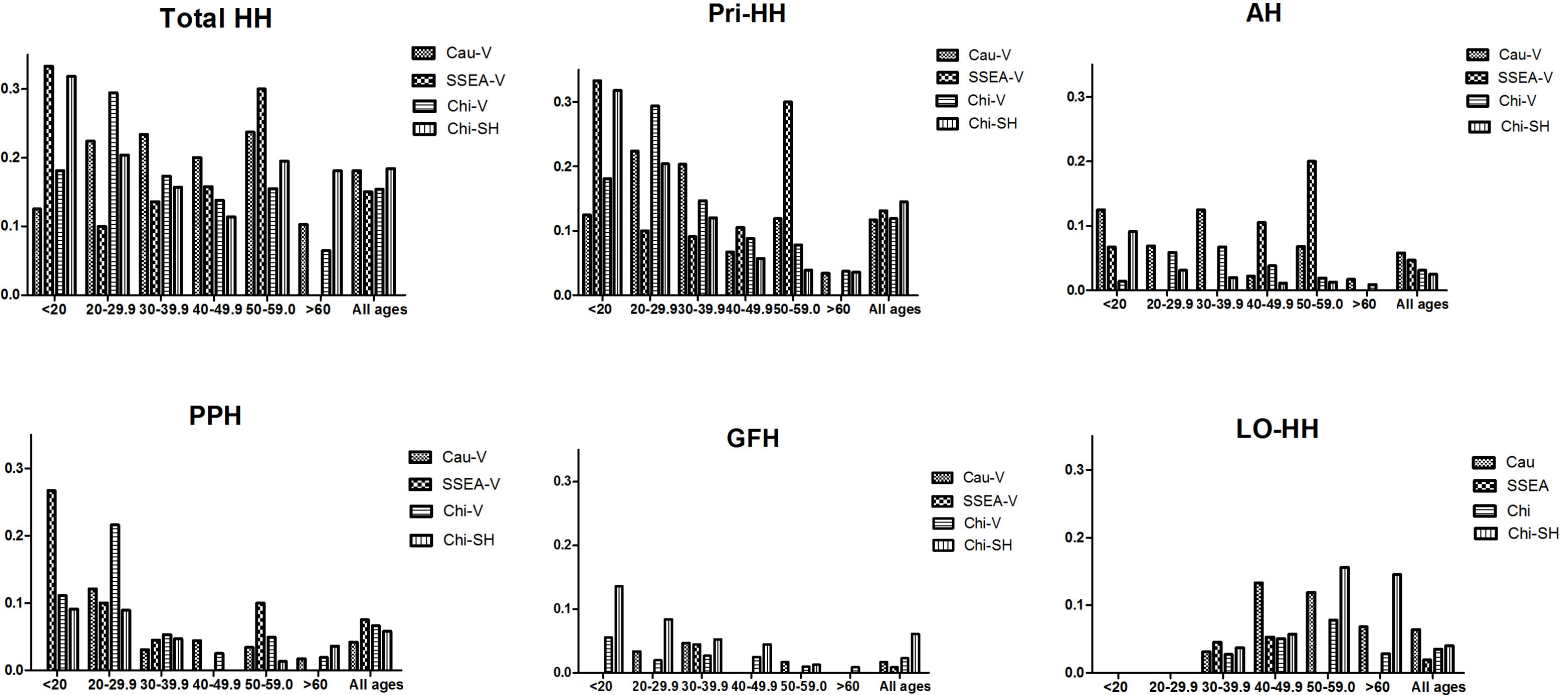
Prevalence of HH and Subtypes in various age groups of dermatology outpatients in Shanghai and Vancouver. Abbreviations: Cau-V: Caucasians in Vancouver; Chi-V: Chinese in Vancouver; SSEA-V: South and Southeast Asians in Vancouver; Chi-SH: Chinese in Shanghai; Total HH: subjects with primary hyperhidrosis and late-onset hyperhidrosis; Pri-HH: primary hyperhidrosis (including AH, PPH, and GFH); AH: axillary hyperhidrosis, PPH: palmoplantar hyperhidrosis; GFH: generalized and facial hyperhidrosis; LO-HH: generalized and facial hyperhidrosis with age of onset at 30 or above.

Although most people with hyperhidrosis seeking medical help are of the age of less than 30 years [9], it is not known if the prevalence of hyperhidrosis remains the same across the entire age spectrum. To analyze this, we stratified the subjects in Vancouver and Shanghai into various age groups and plotted their prevalence of HH and anatomic subtypes (**Fig 1)**. The prevalence of total HH, primary HH, and subtypes are much higher in those younger than 30 years of age. This is decreased in 40’s by more than 50% and remained low for the rest of the age spectrum for palmoplantar HH and generalized and facial HH. For axillary HH, there is a spike of increase in the age group of 50–59, but that was decreased and returned to lower level in those aged 60 or higher. The age distribution of late-onset HH is entirely different, which progressively increases with age from 30 years and above.

Since gender and ethnicity as well as BMI also can influence the prevalence of HH, regression analyses were performed to pinpoint the specific contribution of age on the development of HH. As shown in **Table 5**, the individuals with age <30 years are 2.5 times and 6.4 times more likely to develop hyperhidrosis than those in age groups of 30–60, and over 60 years, respectively. The age trend is the same for primary HH and the three subtypes (AH, PPH, and GFH), decreasing dramatically in prevalence with increasing age (**Table 5**). However, the trend is reversed for late-onset HH, which is two times higher in those >60 years of age compared with that of the younger age groups (p = 0.004).

### Prevalence of HH and presenting diagnoses in dermatology outpatients

To detect any potential influences the primary diagnoses of dermatology patients on the prevalence of HH, multivariate logistical regression analyses were performed, taking into consideration of the impact of age, gender, ethnicity, and BMI. As can be seen in **Table 5**, in general, HH prevalence shows no dependence on the skin concerns the patients presented with, with the exception of psoriasis and vitiligo. Compared with the rest of the subjects, those patients presenting with vitiligo have a 5.1 fold higher prevalence of palmoplantar HH (p = 0.003). Similarly those with psoriasis had a 3.5 fold increased prevalence of developing axillary HH (p = 0.049). The true significance of these observations, however, remains to be confirmed.

## Discussion

The prevalence of primary hyperhidrosis has been the subject of examination by several groups focusing on populations that differ in ethnic compositions, geographical locations, age, and gender. However, the findings vary from one study to the other: 2.8% in the United States [4], 4.4% PPH in Fuzhou, China [10], 16.3% in Germany [5] and 12.8% in Japan [6]. Possible reasons for the wide variation may include differences in study methods, precise definitions used, ethnic composition of the study subjects, age, gender and the environment. Our study examined the prevalence of hyperhidrosis involving multiple ethnicity and multiple centers in the same setting and using the same study method and diagnostic criteria. This showed very similar prevalence of total hyperhidrosis in Shanghai and Vancouver. However, further examinations revealed differences in prevalence according to age, sex, ethnicity, and body weight in dermatology outpatients. In both Vancouver and Shanghai, hyperhidrosis is much higher in those younger than 30 years of age. The observed dramatic decrease of prevalence of primary hyperhidrosis with increasing age is especially prominent for palmoplantar hyperhidrosis. The reasons for the decrease prevalence is not clear, but potentially suggest that primary hyperhidrosis is not a life-long condition. In the majority of the cases, primary hyperhidrosis becomes milder or even resolves with age. This may explain that most people seeking treatments for hyperhidrosis are those younger than 30 years of age. It is at present unknown what physiological /psychosocial aspects of biological age influences manifestations of hyperhidrosis.

In our study, gender has a moderate impact of hyperhidrosis prevalence and presentation. Males are about 20% more likely to develop primary hyperhidrosis than females, especially palmoplantar hyperhidrosis, whereas females are more likely to develop axillary hyperhidrosis and late-onset HH. The late-onset HH may partially be explained by menopause as it reaches the highest incidence at 50–60 years of age.

The hyperhidrosis association with ethnic origin was one of the first to be observed in early hyperhidrosis literature. Cloward asserted that PPH mostly affects South-East Asian [11]. However, this was not formally investigated in a non-biased fashion that involves multi-ethnicity previously. Our study showed that there is a trend for Chinese to have higher prevalence of PPH and GFH, although these differences did not reach statistical significance level. However, Caucasians have much higher chances of developing axillary HH than the Chinese subjects. The reasons for the interracial AH differences are unknown at present.

The impact of obesity or increased body mass index on primary hyperhidrosis has not been investigated previously. The intuition is that increased body weight may cause higher incidences of hyperhidrosis. However, out data showed that, after considering the other contribution factors for primary hyperhidrosis, BMI does not play an important role in the prevalence of primary hyperhidrosis in general, or in specific anatomic subtypes of primary hyperhidrosis. The biggest impact of increased BMI is on the prevalence of late-onset HH, with those having BMI>24.9 more than twice likely to develop late-onset hyperhidrosis than those who are not obese.

We also examined the potential impact of the presenting concerns the subjects have on the prevalence of hyperhidrosis. As shown in multivariate logistic regression analysis (**Table 5**), in general, the presenting diagnoses do not influence prevalence of hyperhidrosis, with potential exception of psoriasis and vitiligo [12]. These observations need to be further confirmed in future studies.

The impact of geographical locations on the prevalence of hyperhidrosis has not been investigated previously. Our study took place in two cities located on opposite sides of the Pacific Ocean. However, the prevalence of primary hyperhidrosis in the Chinese subjects is quite similar, suggesting that geographical locations do not seem to influence development of hyperhidrosis.

In summary, this is a multiethnic and intercontinental investigation of hyperhidrosis prevalence and demographical profile study. It revealed that primary hyperhidrosis is mainly the disease of the young, whereas late-onset hyperhidrosis is primarily the disease of the old age groups. Palmoplantaer hyperhidrosis seem to have similar prevalence in the three ethnic groups investigated whereas axillary hyperhidrosis is highest in Caucasians compared with Chinese and South Southeast Asians. Contrary to common believes, increased body weight does not play a role in the development of primary hyperhidrosis although it is a major risk factor for developing late-onset hyperhidrosis. However, the reverse is true for late-onset HH, which is much more common in those aged 60 or higher.

This study is limited in some aspects; the data were gathered according to patients’ self-reports and the sample size was relatively small in some groups after stratification for, gender, ethnicity and age. Further, being outpatient clinics, the subjects with severe medical conditions affecting mobility may be underrepresented thus not investigated in this study. The female population in the general population is just slightly higher than male [13] whereas in Dermatology Clinics we have much higher representation of females. The proportion of Caucasians in the Vancouver general population is about 40.6% [14], however only 30% of our study population are Caucasians. This difference is due to the referral pattern of the attending dermatologists. However, since the presenting diagnosis of HH was excluded from the HH prevalence calculations, we do not believe this aspect to have major impact on our conclusions.

## Supporting Information

S1 Fig Hyperhidrosis Severity and Negative Impact Scores in Subjects with Hyperhidrosis in Dermatology Outpatients in Vancouver and Shanghai. Hyperhidrosis severity as defined using the following scale: In the last two weeks the frequency of sweating in the absence of thermal or mental triggers every day (score = 3), on more than half of the days (score = 2), one less than half of the days (score = 1, or not at all (score = 0). The impact score is defined using the following scale: In the last two weeks, has excessive sweating negatively affected your daily activities on every day (Score = 3), more than half of the days (score = 2), less than half of the days (score = 1) or not at all (score = 0). Abbreviations: Cau: Caucasian in Vancouver; SSEA-V: South and Southeastern Asians in Vancouver; Chi-V: Chinese in Vancouver; Chi-SH: Chinese in Shanghai; HH: hyperhidrosis.
(TIF)

S1 File Individual database of all participants in Vancouver and Shanghai. (XLSX)

S2 File Patient information questionnaire and examination form. (DOCX)

## Abbreviations

HH: hyperhidrosis
Pri HH: primary hyperhidrosis
AH: axillary hyperhidrosis
PPH: palmoplantar hyperhidrosis
GFH: generalized or facial hyperhidrosis
LO-GFH: generalized or facial hyperhidrosis with onset >30 years of age
BMI: body mass index
C.I.: Confidence Interval
yrs: years old
OR: odds ratio
