# Supplementary figures and images for: Hyperhidrosis Prevalence and Demographical Characteristics in Dermatology Outpatients in Shanghai and Vancouver
Source: PLoS One. 2016 Apr 22;11(4):e0153719. doi: 10.1371/journal.pone.0153719 (PMC4841532; doi:10.1371/journal.pone.0153719)

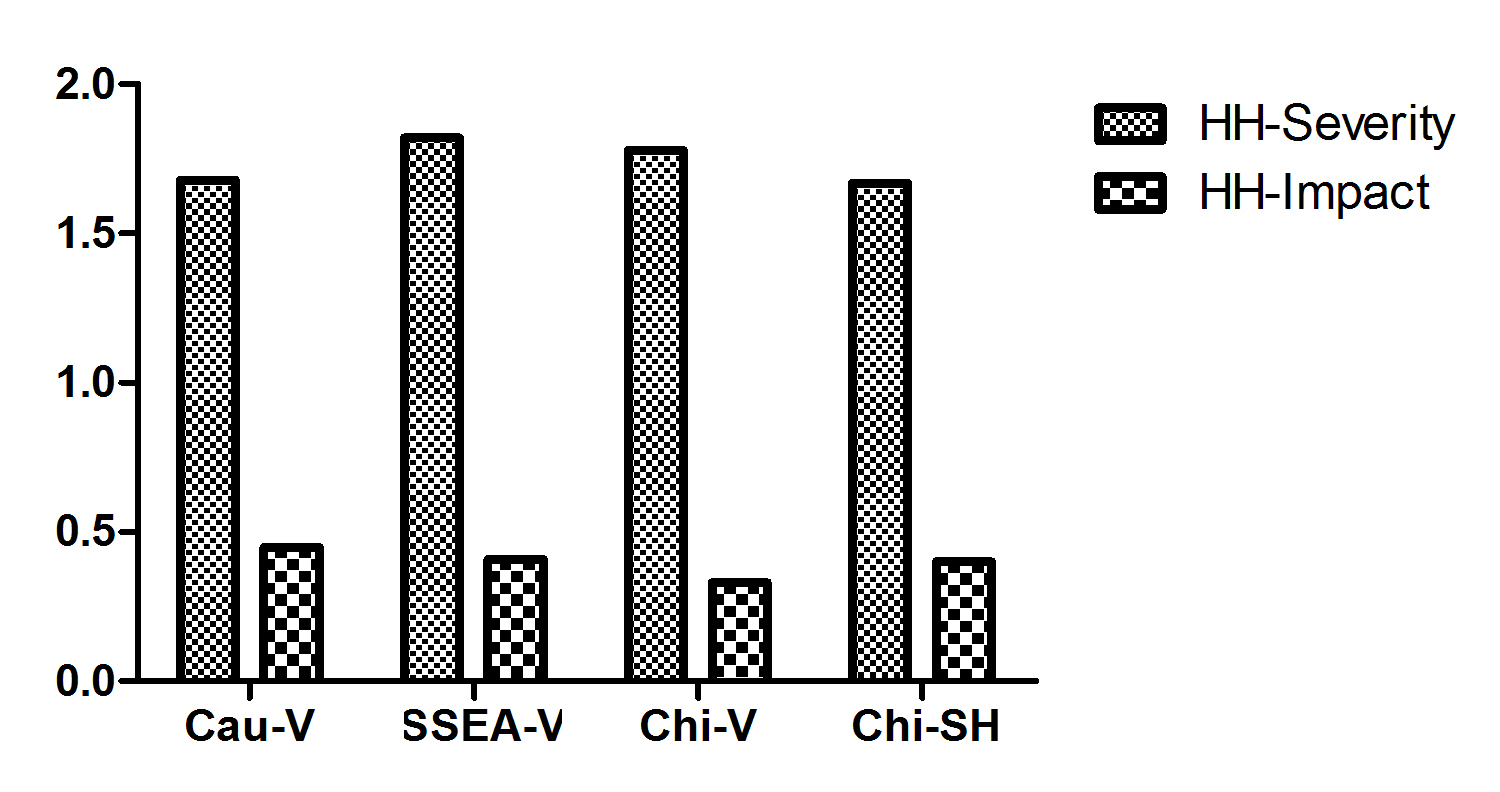

Supplement: S1 Fig — Hyperhidrosis severity as defined using the following scale: In the last two weeks the frequency of sweating in the absence of thermal or mental triggers every day (score = 3), on more than half of the days (score = 2), one less than half of the days (score = 1, or not at all (score = 0). The impact score is defined using the following scale: In the last two weeks, has excessive sweating negatively affected your daily activities on every day (Score = 3), more than half of the days (score = 2), less than half of the days (score = 1) or not at all (score = 0). Abbreviations: Cau: Caucasian in Vancouver; SSEA-V: South and Southeastern Asians in Vancouver; Chi-V: Chinese in Vancouver; Chi-SH: Chinese in Shanghai; HH: hyperhidrosis. (TIF) [file pone.0153719.s001.tif]
