# Supplementary material for: Hyperhidrosis Prevalence and Demographical Characteristics in Dermatology Outpatients in Shanghai and Vancouver
Source: PLoS One. 2016 Apr 22;11(4):e0153719. doi: 10.1371/journal.pone.0153719 (PMC4841532; doi:10.1371/journal.pone.0153719)
Supplement: S2 File — (DOCX) [file pone.0153719.s003.docx]

**Patient information questionaire and examination form**

**Chart No:**____________ **BMI:**_____ **Skin Type**: ____ **Diagnosis**: ________________________

Office Use Only

- - - 1. Today’s date: _______________________ Postal Code: ________________________

1. Ethnic origin: _______________________ Height: ________________________
2. Country of birth:_______________________ Body weight: ________________________
3. Sex: Female (___), Male (___) Age (yrs) ________________________
4. Your current occupation: ________________ Your last occupation (if retired)_____________
5. What is the **main problem** that brought you in today? (List one problem only)

________________________________________________________________________

- How long has the problem been present? ______________________________________
- Does it give you any discomfort or symptoms? (Please use “**✓**" to indicate presence)

Itch ____; Pain ____; Burning ____; Bleeding____; Other:_____________

- What makes the problem worse? ______________________________________
- What makes the problem better? ______________________________________
- What treatments have you tried and how did they work?(List them, use **✓**to indicate effectiveness)

______________________ Better ___ Worse___ No effect ___ Unknown ____

______________________ Better ___ Worse___ No effect ___ Unknown ____

______________________ Better ___ Worse___ No effect ___ Unknown ____

1. Does any family member have the same condition? (If “yes”, list their relationship to you):

_______________________________________________________________________

1. How many people live in your house or apartment? ___________
2. How many toilets are there in your house or apartment? ___________
3. Are there any pet animals in your house or apartment? (If yes, list them below)

_______________________________________________________________________

1. Are you currently taking any **drugs** prescribed by a doctor? (If yes, list the drugs below)

______________________________ ­­­_________________________________________

1. Are you **allergic to any drugs**? (If yes, list the drug allergies below)

________________________________________________________________________

1. Have you been diagnosed with the following medical conditions?(Please use “**✓**" to indicate your answer)

| **Medical Conditions** | **Yes** | **No** | **Details (if “yes”)** | **MD** |
| --- | --- | --- | --- | --- |
| Diabetes |  |  |  |  |
| Hypertension |  |  |  |  |
| Hyperlipidemia |  |  |  |  |
| Thyroid disease |  |  |  |  |
| Depression |  |  |  |  |
| Anxiety |  |  |  |  |
| Bipolar disorder |  |  |  |  |
| Chronic fatigue |  |  |  |  |
| Chronic itch (> 6 months) |  |  |  |  |
| **Skin diseases** |  |  |  |  |
| - Psoriasis |  |  |  |  |
| - Dermatitis (or eczema) |  |  |  |  |
| - Melasma |  |  |  |  |
| - Rosacea |  |  |  |  |
| - Vitiligo |  |  |  |  |
| - Hyperhidrosis |  |  |  |  |
| - Alopecia areata |  |  |  |  |
| - Andro-genetic alopecia   (Male / female pattern hair loss) |  |  |  |  |
| - Skin cancers |  |  |  |  |
| - Other skin diseases |  |  |  |  |
| **Other medical diagnoses** |  |  |  |  |
|  |  |  |  |  |

1. Please tell us how much your skin is exposed to the following factors over the last week:

| **Skin exposures**(on average over the last week) | **Your answers** |
| --- | --- |
| How much time did you spend in the sun? | _____Hours /week |
| How often were your hands washed or exposed to water? | _____Times/day |
| How many packs of cigarettes did you smoke? | _____Packs /week |
| How many alcoholic drinks did you have? | _____Drinks /week |
| How often did you shampoo your hair? | _____Times /week |
| How often did you take showers? | _____Times /week |
| How long did your typical showers last? | _____Minutes |
| How often did you take soaking baths? | _____Times /week |
| How long did your typical baths last? | _____Minutes |
| How often did you use body lotions or moisturizers? | _____Times /week |
| How often did you swim? | _____Times /week |
| How often did you use Jacuzzi / hot tubs/ Sauna ? | _____Times /week |

1. How many blistering sun burns have you had in your lifetime so far? _____Times
2. How many times have you used indoor tanning in your lifetime so far? _____Times
3. Over the past two weeks, have you taken prescription drugs for depression, anxiety or bipolar disorders? Yes___, No___(Please use “**✓**"to indicate your answer)
4. **How often** are you bothered by the following **stress symptoms** over the **last two weeks**?

(Please use to indicate frequency using the scale to the right): 0=not at all

1=less than half of the days

2= more than half of the days

3= nearly every day

| **Symptoms** | **Frequency** |
| --- | --- |
| 1. Having little interest or pleasure in doing things | 0 1 2 3 |
| 1. Feeling down, depressed , or hopeless | 0 1 2 3 |
| 1. a. Having trouble falling or staying asleep,   b. Or opposite Sleeping too much | 0 1 2 3 |
| 1. Feeling tired, or having little energy | 0 1 2 3 |
| 1. a. Having poor appetite,   b. Or opposite Eating too much | 0 1 2 3 |
| 1. Feeling bad about yourself, or thinking that you are a failure, or feeling having let yourself or family down | 0 1 2 3 |
| 1. Having trouble concentrating on things (such as reading the newspaper or watching television) | 0 1 2 3 |
| 1. a. Moving or speaking so slowly that other people could have noticed;   b. Or opposite, being so fidgety or restless that you have been moving  around a lot | 0 1 2 3 |
| 1. Having thoughts that you would be better off dead, or having thoughts of hurting yourself | 0 1 2 3 |
| 1. Feeling nervous, anxious, or on edge | 0 1 2 3 |
| 1. Not able to stop worrying | 0 1 2 3 |
| 1. Worrying too much about various things | 0 1 2 3 |
| 1. Having trouble relaxing | 0 1 2 3 |
| 1. Being so restless that it is hard to sit still | 0 1 2 3 |
| 1. Becoming easily annoyed or irritable | 0 1 2 3 |
| 1. Feeling afraid as if something awful might happen | 0 1 2 3 |
| 1. **Sweating** on hands, armpits or face for no obvious reasons   (That is, sweating even when not feeling nervous, not doing physical activities, and not being in a hot environment) | 0 1 2 3 |
| 1. Sweating at night or during sleep | 0 1 2 3 |
| 1. Your sweating having a negative impact on you | 0 1 2 3 |

1. If you are bothered by **excessive sweating,** how severe is the sweating on the following body sites? (Please indicate the severity: 0 = none,1= mild, 2=moderate, 3 = severe)

Hands ____; Feet_____; Armpits_____; Face_____; Chest/back_____; Other areas_______

1. Would you like to give a saliva sample to help skin research? (It is painless and takes 2 minutes)

Yes______, No______ Your signature: ___________________

Office Use only: Score (1-9)____ Score (10-16) _____ HH Score____________
